# Physiochemical characterization and cytotoxicity evaluation of mercury-based formulation for the development of anticancer therapeuticals

**DOI:** 10.1371/journal.pone.0195800

**Published:** 2018-04-18

**Authors:** Kannan N, Shanmuga Sundar S, Balaji S, Arul Amuthan, Anil Kumar NV, Balasubramanian N

**Affiliations:** 1 Department of Biotechnology, Manipal Institute of Technology, Manipal Academy of Higher Education, Manipal, Karnataka, India; 2 Department of Biochemistry and Molecular Biology, University of Debrecen, Debrecen, Hungary; 3 Department of Chemical Engineering, Anna University, Chennai, India; 4 Dept. of Pharmacology, Melaka Manipal Medical College, Manipal Academy of Higher Education, Manipal, Karnataka, India; 5 Department of Chemistry, Manipal Institute of Technology, Manipal Academy of Higher Education, Manipal, Karnataka, India; Yeungnam University, REPUBLIC OF KOREA

## Abstract

**Background:**

The present study is aimed to evaluate the physiochemical properties and cytotoxicity of mercury-based formulation for the development of anticancer therapeuticals.

**Methods:**

The elemental and morphological features of the formulation were characterized by FE-SEM, XPS and EDS. The described formulation was evaluated for its cytotoxicity on Hek293 and MCF7 cell lines using MTT assay to study the *in vitro* effects. The *in vivo* developmental toxicity was also studied on zebrafish embryos and the lethal concentration (LC_50_) values were calculated as per the OECD regulations.

**Results:**

The elemental and morphological characterizations confirmed the presence of mercuric compounds. The particles were spherical and stable with the size ranges between 20 and 80nm. Although the PK formulation contains mercurials it was very effective only to cancerous cells (MCF-7) and it is less toxic to normal cells (HEK 293). The *in vivo* assessment of developmental toxicity on zebrafish embryo confirmed the safer dosage of 100μg/ml. However, a higher dosage of 1mg/ml led to the malformation of embryos such as pericardial, tail and yolk sac edema.

**Conclusion:**

The physiochemical characterization of PK formulation confirmed the presence of HgS. The results of both *in vitro* and *in vivo* studies showed that the formulation is less toxic. Although the test sample contains mercurials it was very effective against cancerous cells (MCF-7) and it is less toxic to normal cells (HEK 293).

**Future studies:**

Further studies on effectiveness of the formulation along with inflammatory response in mice models are to be conducted.

## Introduction

Metals and minerals are utilized in medicine for the treatment of many diseases including cancer [1, 2]. These are widely used in traditional medical systems practised in Asian countries such as China [3], India [4], Japan [5], Nigeria [6], Srilanka [7] and Tibet [8]. Metallic and organo-metallic compounds are also accessible in Western medicine soon after the discovery of cisplatin in 1960 by Barnett Rosenberg [9, 10]. However, there are major concerns due to their toxicity and unintended side effects. At the same time there are also several reports on beneficial effects [11].

Heavy metals such as arsenic [12, 13], cadmium [13, 14], chromium [15], lead [16], mercury [17] and nickel [16] are scarcely used in Western medicine due to its toxicity [1], whereas it is quite evident that the ancient traditional medical system use cinnabar (mercuric sulfide) to treat specific ailments. One such Indian medicine ‘*Pattu Karuppu (PK)’*, a mercury-based *Siddha* formulation prepared by the combination of acidic and alkaline substances that has a natural affinity for each other [18].

PK formulation is rich in mercuric sulfide (HgS) and it is used in treating dysmenorrhoea, amenorrhoea and delirium [19]. Mercury is well known for its curative effect and the presence of sulfur neutralizes the toxicity and also enhance its therapeutic effect [20]. However, the effectiveness of PK in treating cancer has not been studied. Hence, the objective is to investigate the physicochemical properties of this formulation as well as to evaluate its cytotoxic effects both *in vitro* (Hek 293 & MCF-7 cell lines) and *in vivo* (developmental toxicity on zebrafish embryos).

## Materials and methods

### Metal formulation

The HgS formulation, *Pattu Karuppu* (Batch Number: SII-019) was obtained from IMCOPS, Chennai (The Indian Medical Practitioners Co-operative Pharmacy and Stores) Ltd, Tamilnadu, India.

### Characterization of the formulation

The X-ray powder diffraction (XRD) pattern of the formulation was collected using Rigaku MiniFlex600 (Cu-Kα, λ = 1.5406 Å) operated at 40 kV, 15mA (step scan mode, 0.05°/0.5 sec, 1 sec/step). The EDS and XPS were a destructive method used to detect the elements at surface level. The elemental composition on the surface level was carried using X-ray Photoelectron Spectroscopy (XPS) using Carl Zeiss, Oberkochen. Germany equipped with Ultra 55 FESEM with EDS. The spectra were acquired in an ultra-high vacuum with Al Kα excitation at 250W using energy dispersive X-ray microanalysis system (EDS, Oxford Instruments, High Wycombe, UK). Inductively coupled plasma optical emission spectrometry (ICP-OES, Perkin-Elmer 5300 DV, Wisconsin, US) was used to quantify the elements in low concentration. The micrographs were recorded using field emission scanning electron microscopy (FESEM) (Carl Zeiss AURIGA- CrossBeam, Oberkochen. Germany). A pinch of the sample was mounted on clean stubs and sputter coated with gold under moderate vacuum (10^−3^ bar) for about five min and placed inside the chamber (airtight) for analysis. Stability and particles dispersity in aqueous medium was characterized by Zetasizer (Malvern Instruments, WR14 1XZ, UK). The surface area of the formulation was analyzed based on adsorption of nitrogen on particle surface using BET isotherm (Smart Instruments, Maharashtra, India).

### *In vitro* cytotoxicity assay

#### Cell viability assay

The effect of the test sample was analyzed using Hek 293 (embryonic kidney-derived) and MCF-7 (breast cancer) cell lines, which are procured from National Center for Cell Science (NCCS), Pune, India. The culture was maintained in DMEM Medium supplemented with 10% FBS. The sample stock solution was prepared by dissolving 1mg/ml. The MTT assay was performed with the seven different concentrations from 10 μg/ml to 70 μg/ml along with control. The cell viability was done in triplicates and analyzed the effective concentration by spectrophotometer at 545nm [21].

#### Trypan blue exclusion test

The cell morphology of the test and control was investigated using Trypan blue dye exclusion test. The trypan blue dye exclusion test was used to detect the viable and non-viable cells. The cells were stained using 0.2% Trypan blue solution for 10 min and fixed with Trypan blue dye at 4°C. The slides were rinsed with phosphate buffered saline and observed under a light microscope at 20x magnification.

### *In vivo* toxicity in zebrafish model

The *in vivo* toxicity in zebrafish model was carried with the approved protocol and guidelines by the Institutional Animal Ethics and Biosafety Committee of Manonmaniam Sundaranar University, Tirunelveli, Tamilnadu, India (Ethics committee approval number: MSU/Ethical/2012/05). Zebrafish were maintained in 30L tanks at 28°C with 14:10 hour light: dark cycles. The eggs were collected from the bottom of the tank and rinsed with running water for three times. The eggs were inoculated into the embryo rearing solution (ERS) (composition in mM: MgSO_4_, 0.16; KCl, 0.17; CaCl_2_, 0.4; NaCl, 5) and incubated in dark at 28°C [22]. The embryos were quality checked at eight hours post fertilization (hpf) using dissection microscope, the damaged embryos were removed. The 48 hpf embryos were treated with various concentrations of formulation in a 48 well plate with 10 embryos in each well containing 1 ml of embryo rearing solution and 1% DMSO. The embryos used as control were also maintained in the presence of 1% DMSO. The changes in the developing embryos and larvae were observed for 96 hours. The abnormalities during organ development were monitored under a light microscope (Coslab, India). The lethal concentration (LC_50_) was calculated as per the OECD regulations [22]. The heartbeat count was recorded to evaluate the cardiac assessment. The three days post fertilization (dpf) embryos were treated with different concentration for four hours. The heartbeat rate (HBR) was recorded for 15 sec in the attached camera at 10X objective lens magnification and processed for heart rate using Adobe premiere 6.5 and ImageJ (NIH, Maryland, US). Embryos have anesthetized with 0.02% tricaine (Sigma, India) to measure the HBR.

## Results and discussion

### Morphological characterization

**Fig 1:**
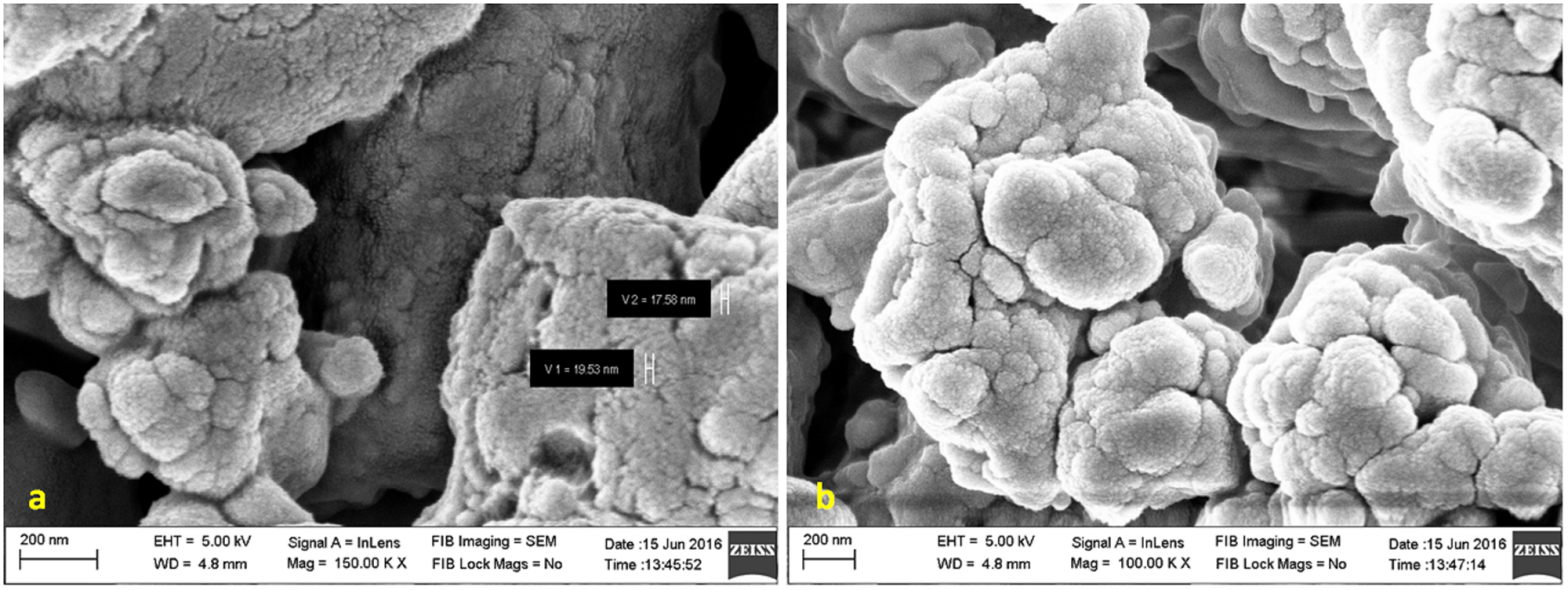
Morphology of PK formulation displaying (a) porous grains (b) broccoli-like pattern.

The surface morphology of the PK formulation was analyzed with FESEM. The individual particles were mostly spherical and the average grain size was in the range of 10-100nm. The particles were not uniformly distributed and have irregular grain boundaries (pore formation, Fig 1) due to higher surface energies. The heat treatment & mechanochemical grinding during the initial preparation of the particles could have resulted in the enhanced surface energy which may lead to agglomeration and is in agreement with Wadekar et al. [23]. Different shapes of particles are attributed to the polydispersity in accordance to Mukherjee et al. [24]. It was confirmed by FESEM that the formulation existed as agglomerated spheres analogous to the broccoli-like pattern (Fig 1).

**Fig 2:**
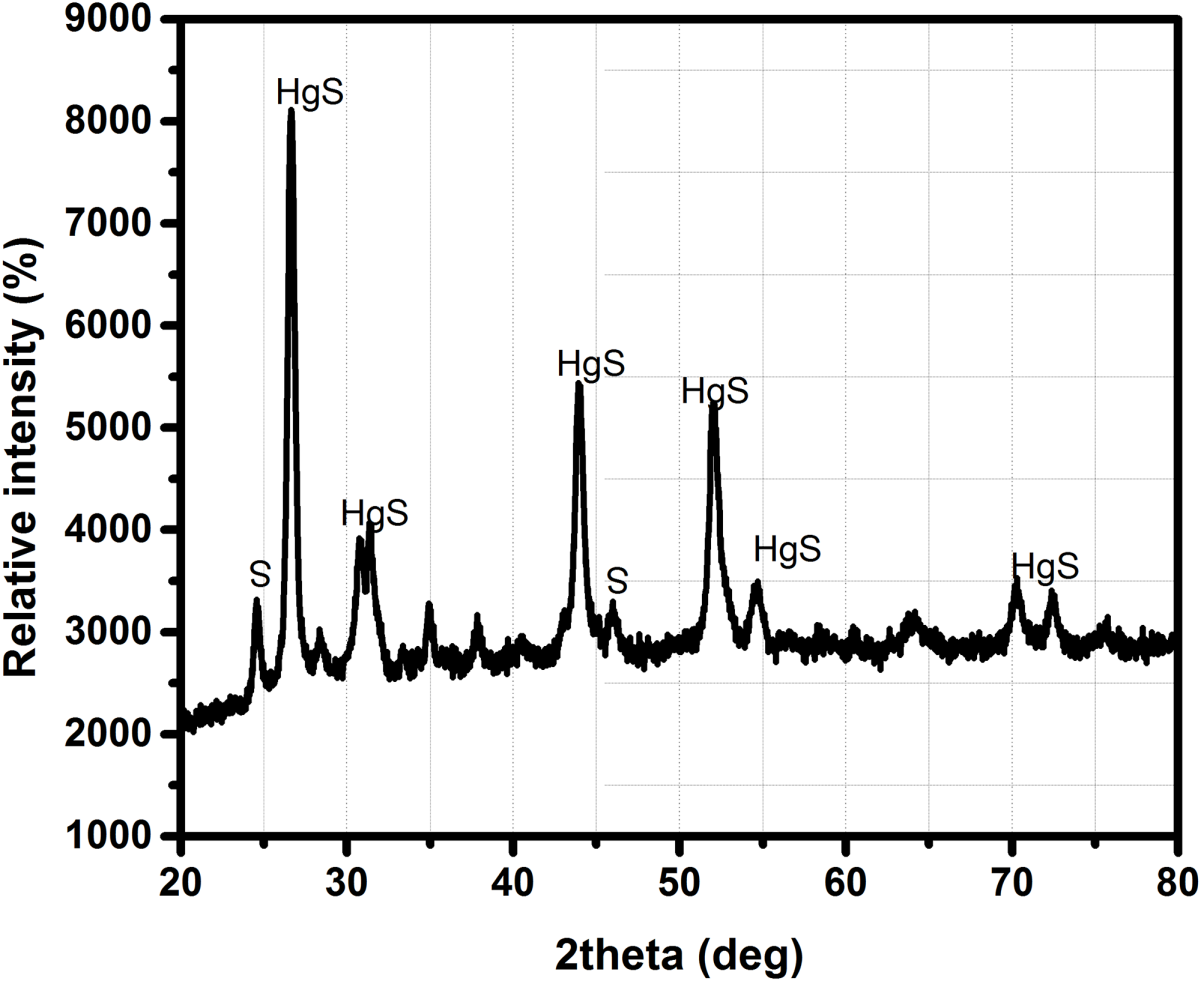
The XRD pattern of PK formulation.

To confirm this further the particles were tested using XRD and zeta analyzer. The diffractogram obtained from the XRD was identical to that of the HgS (Fig 2). The major peaks were corresponding to HgS (JCPDS file no: 19–0798 and 73–1593) with the crystallographic planes of (111), (200), (115), (214), (220), and (411). The broadened signals of the patterns may be due to the destabilized dimensions of the particles [25]. The crystalline size of the particles was calculated using Debye-Scherrer equation, the size of full-width half maximum at 26.53° (2θ) is approximately 88nm.

Particle distribution was carried out in the aqueous dispersion medium and the zeta potential of the formulation was found to be -32.20mV when observed for 100% area under the curve with the width of 87.16 nm. The results showed that the formulation was stable. The zeta potential values were between -23 and -37 for the reported HgS based formulations [24, 26]. The average hydrodynamic diameter of the β-HgS aggregates was reported to be 296.2 ± 22.5nm. The observation of such large aggregates may be due to the sample preparation and drying [27]. The results of XRD and FESEM also supplement this information. This justifies the presence of negatively charged particles in agglomerated form.

The nitrogen adsorption in BET analysis showed that the results of the formulation were 1.41m^2^/g which corresponds to the average particle diameter of approximately 50nm. The surface area analysis confirmed the aggregation. This is in agreement with Xu and Carraway [28].

### Elemental characterization

**Fig 3:**
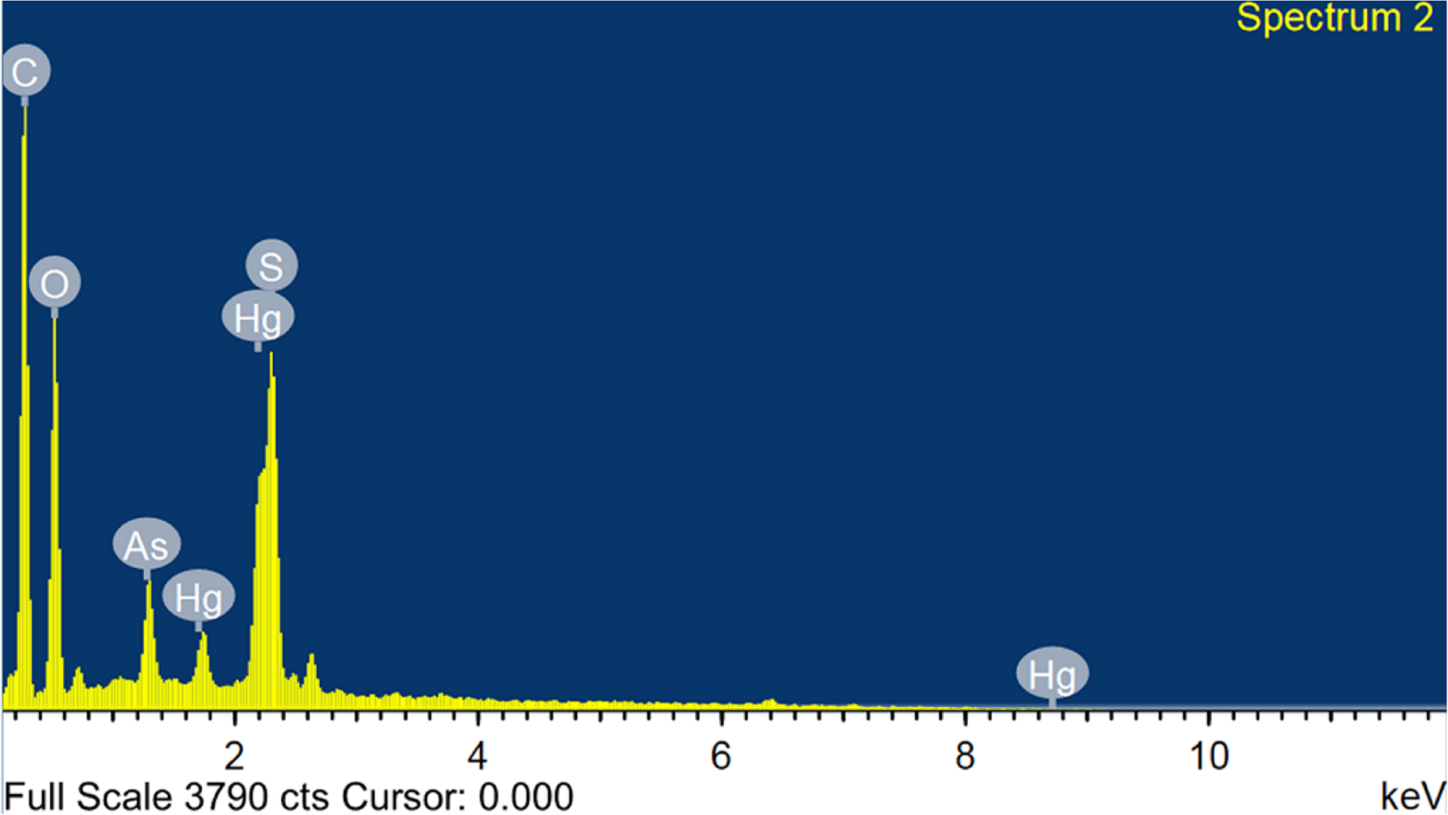
EDS spectrum of PK formulation.

The quantitative elemental analysis was carried out using EDS, ICP-OES and XPS. The EDS spectrum observed for the selected portion is shown in Fig 3. The spectrum confirms the presence of abundant C (26.336%) and O (52.66%) with other expected elements like Hg (5.66%), As (2.58%), and S (12.86%). It is noteworthy, that the presence of C in the formulation is due to the plant extract used in the purification process and expected from the ash content of the formulation. The organic moieties bound to the metal form the organometallic complex and can transform the metals into biocompatible while withstanding high temperatures [29]. It was reported that site-specific action of metal alone is severe but the organometallic complex can quickly pass through the tissues than the other forms [30]. The appearance of O (52.66%) peak, which is expected to bind on the surface and also oxidizes the surface [26, 31]. The results are supported by XRD analysis, which identified the HgS in the sample.

**Fig 4:**
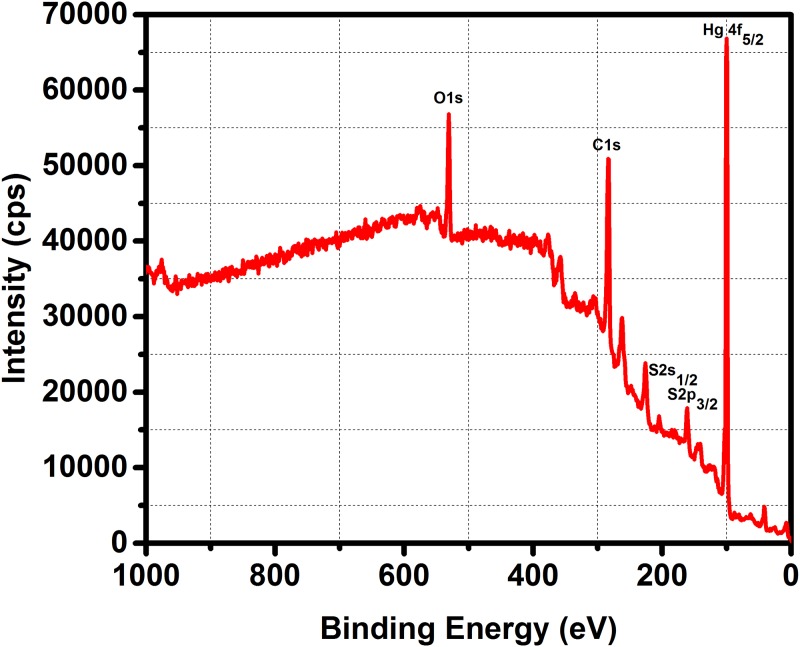
XPS analysis of PK formulation.

The results of ICP-OES revealed that the elements (in ppm) such as Hg (1.522), As (1.577), and S (1.950). The concentration is in agreement with the synthesized cinnabar [32]. The ore of mercury (cinnabar) contains approximately, 84–85.2% of Hg and 14.8–16% of S. The elemental composition on the surface level was investigated by XPS. The XPS spectrum of the test sample is shown in Fig 4. The measured binding energies were obtained by a reference spectra of C1s at 285.20 eV. The sharp peaks at two locations, 100.60 and 104.60 eV were assigned for the Hg (4f) binding energy. The peaks at 161.72 and 162.10 eV are due to S2p_3/2_ and S2p_1/2_ transitions and the S2s_1/2_ was observed at 225.40 eV. The other peaks at 284.61 and 531.0eV were matched with the C1s and O1s respectively. The presence of C1s was attributed to the herbal treatment (trituration) during the preparation of the formulation. The occurrence of oxygen binding energies may be due to the surface adsorption molecules. These binding energy values are well supported by the reported data [33]. Moreover, there are no other peaks in XPS spectrum and thereby it confirms the formulation has Hg and S, probably in HgS phase. The results converge the chance of metal-herbal binding (cation) in the initial stages of pre-treatment may enhance the efficacy of the formulation. The measurement of Hg and S peak area (raw area, cps) was approximately 1:1, which is very close to the composition of HgS. Presence of C and O, which are the building blocks of the organic molecules, on the surface of the formulation by XPS supports the idea of the coating of organic molecules on the surface of the metallic compounds. Thus the XPS analysis also confirms the presence of HgS in the sample. The physiochemical characterization affirms that the formulation is composed of HgS. The PK sample was found to have stable and spherical (porous) particles with size ranges between 20-80nm. The negatively charged, nanosize and relatively high surface area of the particles were used to evaluate its biological action.

### *In vitro* cytotoxicity assay

**Fig 5:**
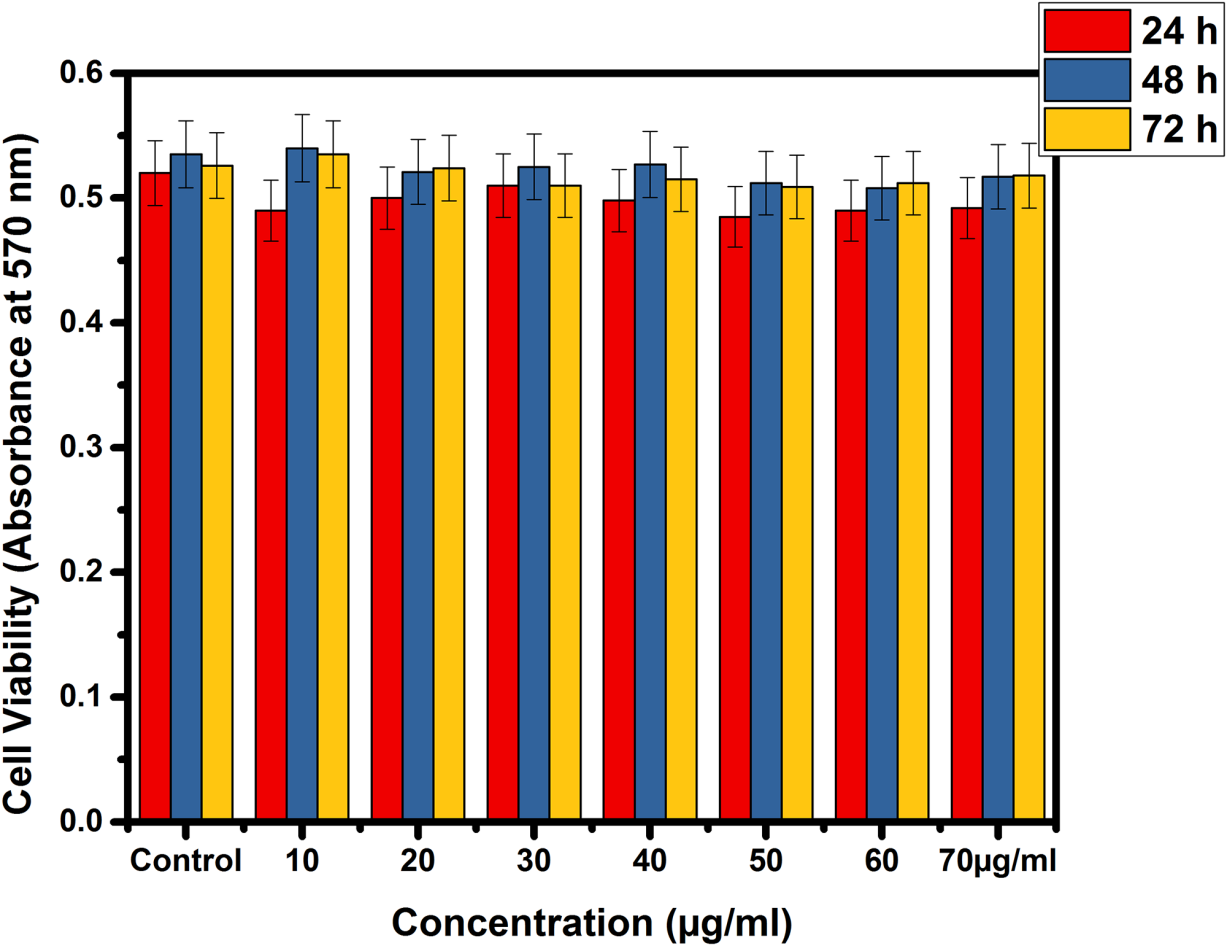
Time course profile of cell viability for Hek293.

**Fig 6:**
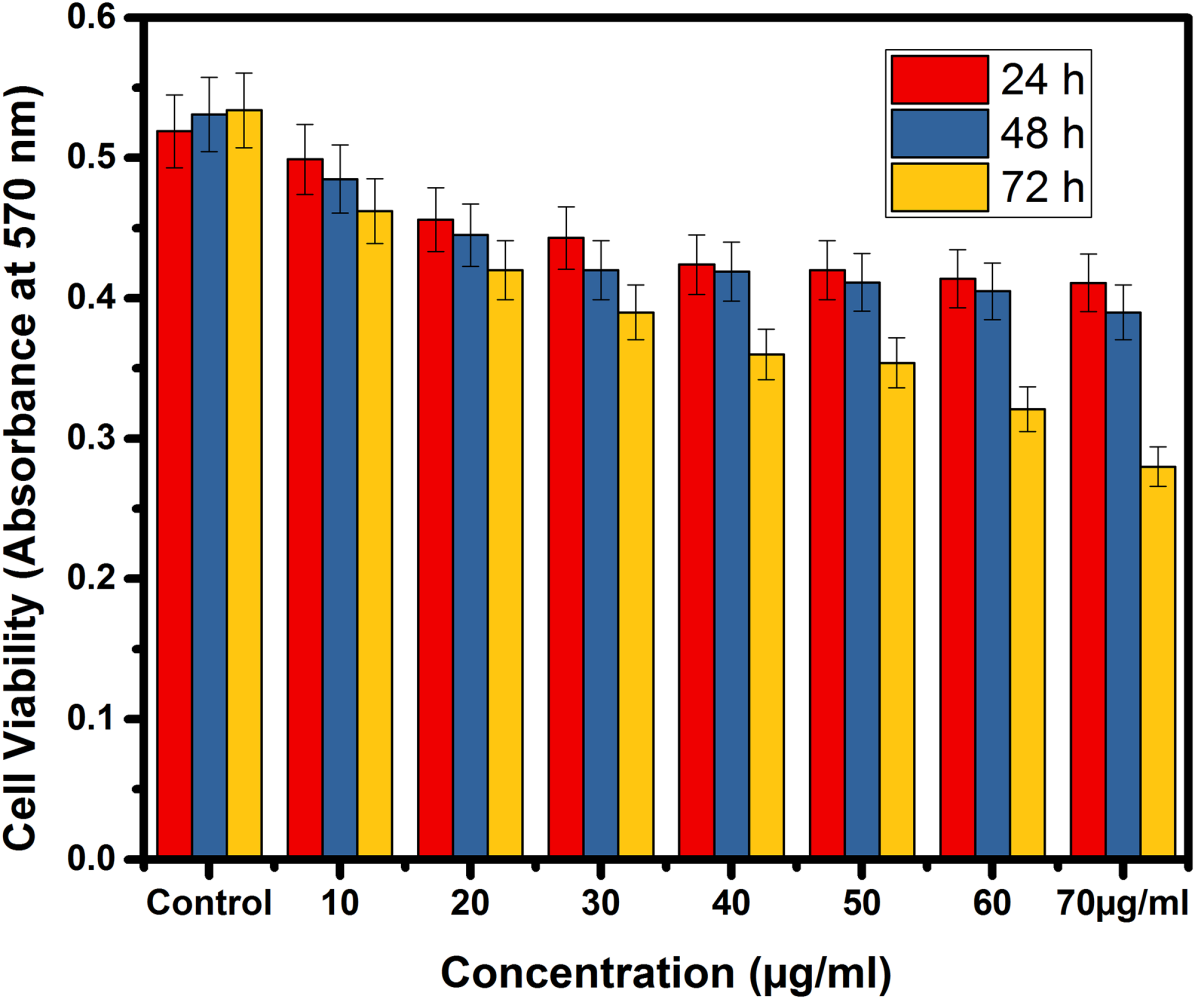
The cell viability of MCF-7.

The cell viability of the sample was determined by MTT assay (Figs 5 & 6). It was observed that live cells with active mitochondria could cleave the MTT resulting in the formation of formazan and produce positive signals, whereas the dead cells will exhibit only negative signals [34]. The sample showed a tendency to slightly inhibit the proliferation of normal cells in a time-dependent manner. The inhibition was higher in the case of immortal cell lines. The response observed as the concentration increase from 10 to 70 μg/ml the percentage viability of the cell has not changed to a greater extent in the case of normal Hek 293 cell lines but decreased in the case of MCF-7 cells (Figs 5 & 6). On the one hand, Hek 293 had an IC_50_ value of 35.64 μg/ml on 24 hours (i.e., greater than 20μg/ml), on the other, IC_50_ of the MCF-7cells were lesser than 20μl (i.e., 19.61 μg/ml). It is evident from the MTT assay that the formulation was more effective on the immortal cells than normal cells. This is due to increased IC_50_ values for Hek 293 and decreased IC_50_ values for MCF-7cells (Figs 5 & 6) both are time-dependent (up to 72 hours). The cytotoxicity is inversely correlated with IC_50_ this is in agreement with Florento et al. [36] and Ulukaya et al. [35].

**Fig 7:**
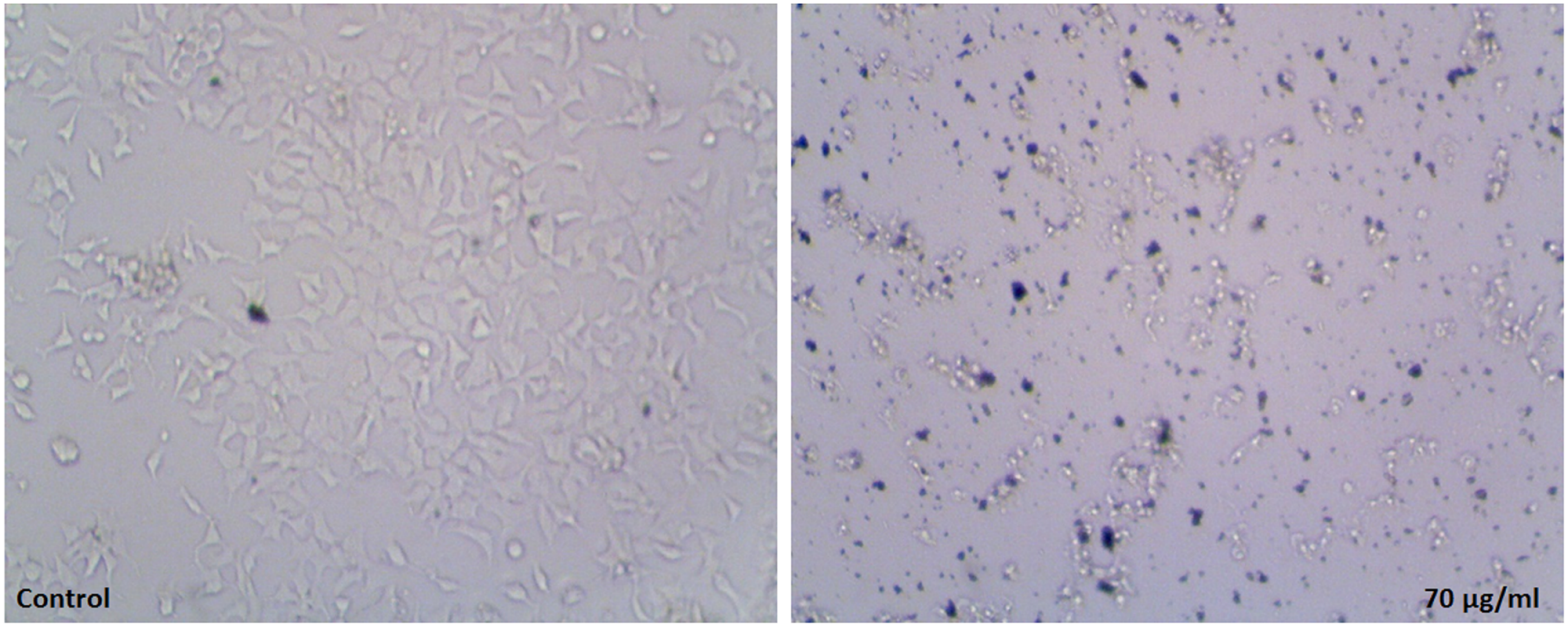
Trypan blue stained Hek293 cells after treatment with control and higher concentration.

**Fig 8:**
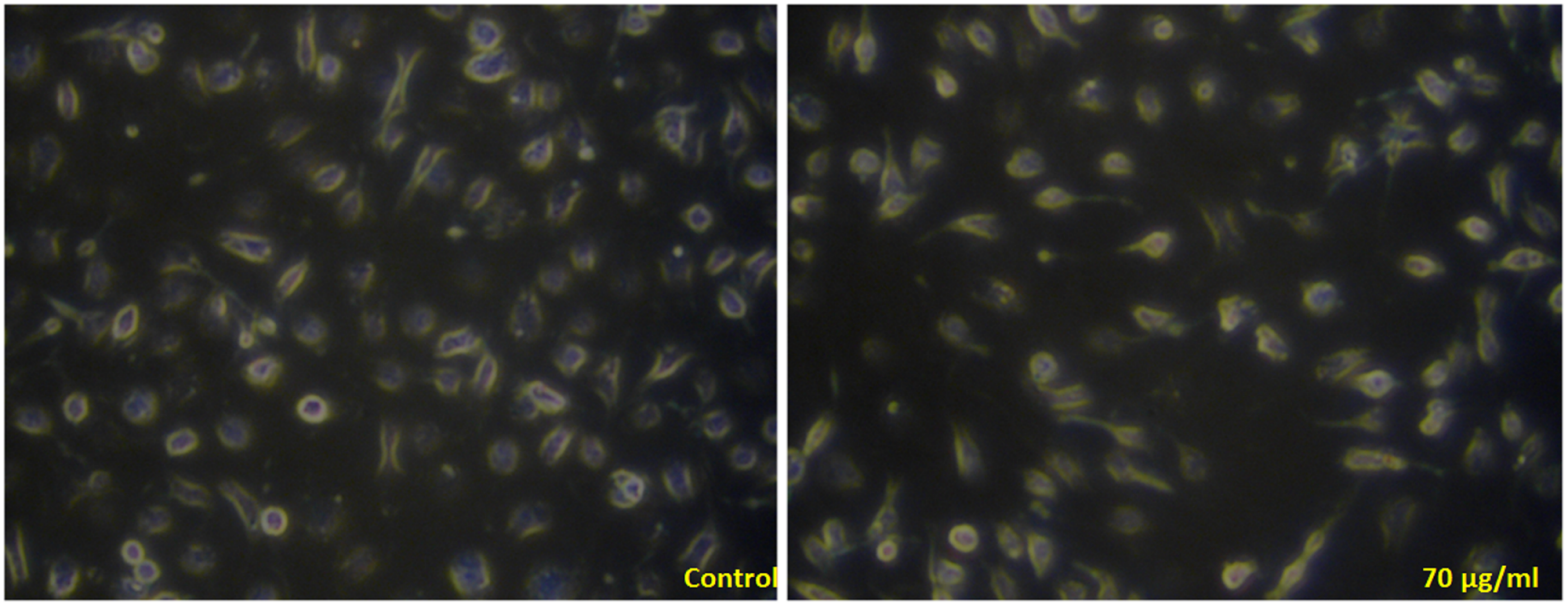
Trypan blue stained MCF-7 cells after treatment with control and higher concentration.

The results of trypan blue staining distinguished viable and non-viable cells. This confirmed the effect of PK sample on immortal and normal cell lines which was on par with Florento et al [36]. It is also evident that the live cells were more in Hek293 compared to MCF-7 cell lines (Figs 7 & 8). The damaged cells and cell debris were observed in MCF-7 whereas the intact cell membrane was observed in Hek293 [37, 38].

### *In vivo* developmental toxicity on zebrafish model

**Fig 9:**
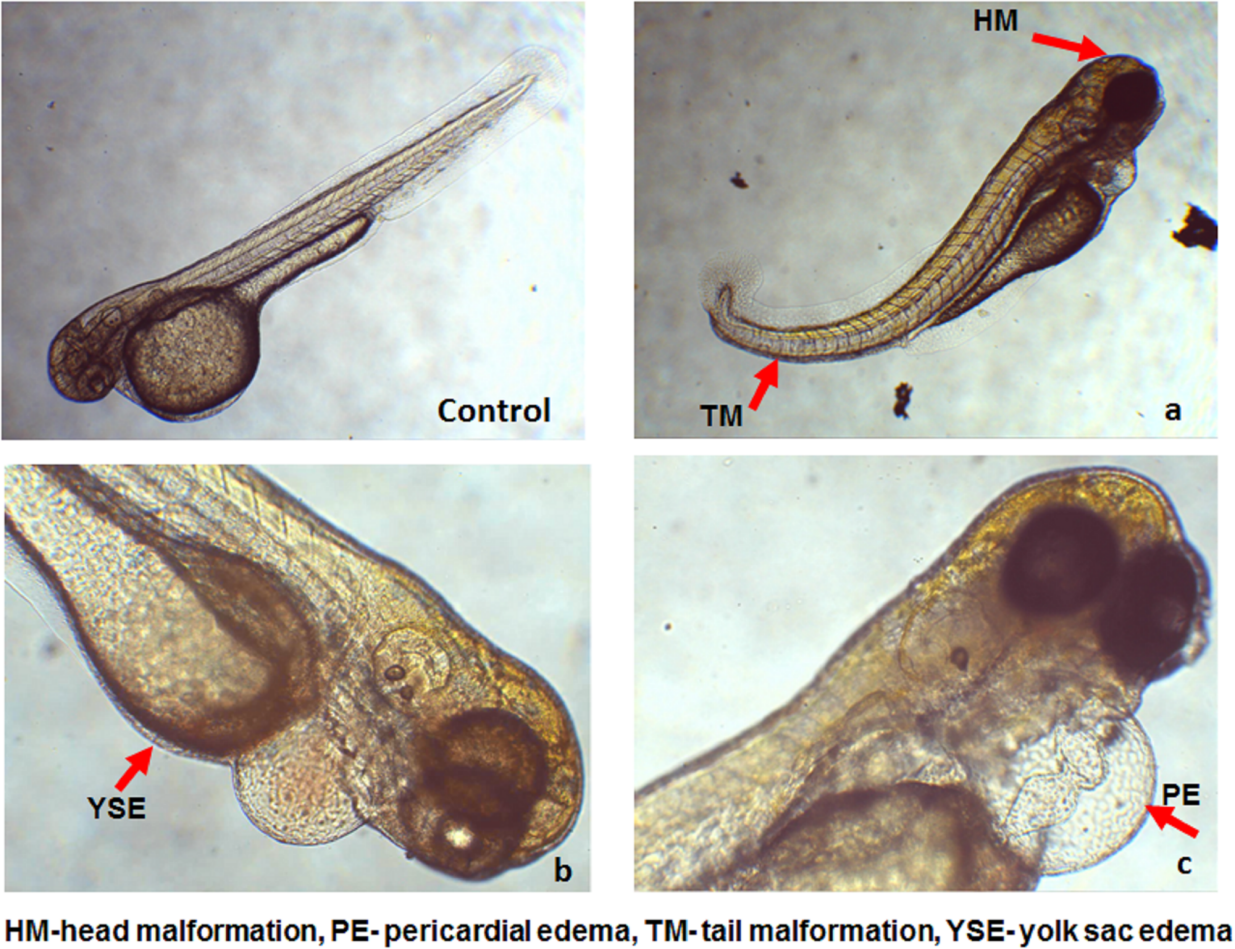
Deformities in zebrafish embryo development at 1 mg/ml concentration (a) head and tail (b) yolk sac edema and (c) pericardial edema.

The safer dosage level of the formulation was evaluated *in vivo* on zebrafish embryos (Fig 9). The mortality and heartbeat of the embryos were observed for 96 hpf with different concentrations from ng to mg (1, 10, 100 ng/ml), (1, 10, 100 μg/ml), and (1, 10 mg/ml). The maximum nonlethal concentration (MNLC) of the sample was found to be 100 μg/ml. It was observed that there is no significant difference in mortality of embryos and larvae with the MNLC. The LC_50_ of the sample is 1mg/ml and dose-time dependent toxicity was observed at 1 mg/ml. The results of 1 mg/ml showed the malformation of embryos (pericardial, tail and yolk sac edema) [39].

The heartbeat rate (HBR) assessments of embryos were surveyed at 4 hpf. There were no significant changes in the HBR up to 1mg/ml. The HBR of embryos was 44 beats/min in control whereas it was observed to be below 40 beats/min for 10 mg/ml in treated embryos. The sample treated embryos had taken 11 frames to complete one cardiac cycle and the control had taken 20 cycles to complete one cardiac cycle. The exposures of high concentration may lead to arrhythmias [39]. The phenotypic deformity analysis revealed thrombosis of blood cells in the cardiac chamber at the tested concentrations. These results could confirm that the safe formulation concentration for the sample is 100μg/ml.

## Conclusion

Taken together the results of FESEM, XRD, XPS, EDS, ICP-OES, zeta potential and BET, elucidated the morphological properties such as size (20-80nm), shape (broccoli-like), surface charge (-32.20mV) and surface area (14.1 m^2^/g) of PK formulation. The elemental analysis determined the preponderance of HgS. Further, *in vitro* and *in vivo* cytotoxicity assays showed that the sulfide form of mercury even at the concentration of 100μg/ml is safer in normal cells. Hence, this formulation is different from toxic mercurials. Future studies on mouse models are underway to pave the way for the development of anticancer therapeuticals.
