# Correction: Physiochemical characterization and cytotoxicity evaluation of mercury-based formulation for the development of anticancer therapeuticals

**DOI:** 10.1371/journal.pone.0200035

**Published:** 2018-06-26

**Authors:** N Kannan, S Shanmuga Sundar, S Balaji, Arul Amuthan, NV Anil Kumar, N Balasubramanian

The first, second, third, fifth, and sixth authors’ names appear incorrectly. The correct author byline is: N Kannan, S Shanmuga Sundar, S Balaji, Arul Amuthan, NV Anil Kumar, N Balasubramanian.

The correct citation is: Kannan N, Shanmuga Sundar S, Balaji S, Amuthan A, Anil Kumar NV, Balasubramanian N (2018) Physiochemical characterization and cytotoxicity evaluation of mercury-based formulation for the development of anticancer therapeuticals. PLoS ONE 13(4): e0195800. https://doi.org/10.1371/journal.pone.0195800
